# Incidence and predictors of mild cognitive impairment (MCI) within a multi-ethnic Asian populace: a community-based longitudinal study

**DOI:** 10.1186/s12889-019-7508-4

**Published:** 2019-08-22

**Authors:** Norlela Mohd Hussin, Suzana Shahar, Hanis Mastura Yahya, Normah Che Din, Devinder Kaur Ajit Singh, Mohd Azahadi Omar

**Affiliations:** 1 Centre of Healthy Aging and Wellness, Faculty of Health Sciences, Universiti Kebangsaan Malaysia, 50300 Kuala Lumpur, Malaysia; 2 Centre of Rehabilitation Science, Faculty of Health Sciences, Universiti Kebangsaan Malaysia, Jalan Raja Muda Abdul Aziz, 50300 Kuala Lumpur, Malaysia; 3 Institute of Public Health, Ministry of Health, Jalan Bangsar, Federal Hill, 59000 Kuala Lumpur, Malaysia

**Keywords:** Incidence, Predictors, Older adults, MCI

## Abstract

**Background:**

Limited information is available from longitudinal studies regarding the predictors and incidence of MCI in older Asian adults. Thus, a community-based longitudinal study was conducted to determine the incidence of MCI among multi-ethnic older adults in Malaysia. The role of health and lifestyle as predictors of MCI was also examined.

**Methods:**

Analysis of data obtained from the *Towards Useful Aging* (TUA) study (2014–2016), wave 1 (baseline) and wave 2 (1½ years of follow-up) was conducted. For the baseline, comprehensive, interview-based questionnaires were administered to 1227 subjects who were 60 years old and above. MCI is a unique transitional state between normal ageing and dementia. MCI characteristics include a decline and disturbance of cognition, minimal impairment of complex activities, ability to perform regular daily functions, and absence of dementia. The incidence of MCI was assessed using comprehensive neuropsychological batteries. The study then performed a logistic regression analysis to examine the effect of each possible predictor of MCI. This analysis began with univariate analyses and a separate review of the effect of every variable. Binary logistic analyses followed hereafter.

**Results:**

During the follow-up after 1½ years, 179 (14.6%) of the participants who did not exhibit MCI at baseline were observed to have developed MCI. Among the participants who did not exhibit MCI at baseline, the incidence rate was 10.5 per 100 person-years. Male sex and lack of engagement in mental activities were predictors of MCI among participants without MCI at baseline.

**Conclusion:**

After the 1½-year follow-up, the incidence rate for MCI was considerably high among the respondents. Being male and being less engaged in mental activities were predictors of the occurrence of MCI. Mental activities need to be promoted for the prevention of MCI incidence among older adults.

**Electronic supplementary material:**

The online version of this article (10.1186/s12889-019-7508-4) contains supplementary material, which is available to authorized users.

## Background

MCI is distinct from dementia, in which the latter is defined as the extent of cognitive decline that affects daily function while the functional activity remains intact [1]. The degree of decline in cognition ranges from MCI and age-related cognitive decline to severe dementia, such as Alzheimer’s disease. The decline in cognition is known to be multicausal, with MCI not necessarily progressing to dementia in all cases. MCI have mainly been evaluated through the neuropsychological testing of the aforementioned skills over varying time frames. However, MCI contributes minimally to the pathological changes associated with Alzheimer’s disease [2].

In older adults, MCI has been observed to be one of the causes of increased risk of mortality and morbidity around the world. Furthermore, it is significantly taxing to the affected individuals, the people caring for them, and society as a whole [2]. A study involving caregivers reported that strong cultural and spiritual beliefs influence the management of behavioural and psychological symptoms in people with dementia [3]. An ageing population coupled with late-life MCI raise the costs for individuals and society. The causes of this problem, however, remain unclear [4]. MCI has also been linked to an increased risk of dementia, the development of comorbid diseases, and increased rates of mortality and hospitalisation in older adults [5].

A few cross-sectional studies have reported an increase in the prevalence of MCI among older Asian adults [6, 7]. However, limited information is available from prospective studies about the incidence rate and associated risk factors of MCI. Molecular chaperones such as cellular stress biomarkers in blood will reflect the degree and extent of brain damage, thus providing a useful tool for the diagnosis, prognosis, and early treatment of Alzheimer’s disease [8]. Hypertension or high blood pressure has consistently been found to be the most associated health risk factor for MCI [9, 10]. Other factors associated with increased risks of MCI include diabetes mellitus [11, 12] and current-smoking status [13]. In terms of psychosocial issues, MCI has been consistently associated with depression [14–16]. Another study showed that a higher risk of depression among single (unmarried, divorced, and widowed) than married elderly [17]. Hence, being recently single might lead to feelings of isolation and loneliness, which could lead to mental [17]. However, there have been inconsistent findings on the relationship between MCI and living alone [2].

Moreover, increased participation in cognitive enhancing activities in later life is associated with a reduced risk for MCI [18–20]. Preliminary evidence has suggested that physical activity [21, 22] and other leisure activities (e.g., religious services, club membership, gardening, or painting) [18, 23] are positively associated with the preservation of cognitive function. Most Malaysians are not active, and only a small percentage participate in regular and adequate physical activity [24]. The prevalence of physical inactivity among Malaysian men is 37% [25]. Health-promoting strategies that increase awareness, knowledge, skills and motivation related to physical activity are required [26].

Furthermore, a diet high in vegetable intake and low in saturated fats has also been linked to a lower risk of MCI [2]. However, there is an inconsistent association between the intake of vitamins C, B12, and E and MCI [27]. A cohort study illustrated that the Mediterranean dietary patterns lowered the rate of MCI among 3790 older adults [28]. Furthermore, another cohort study with 3054 subjects indicated that individuals who have healthier diets (whole grain, vegetables, fruits, fish, fresh dairy products, legumes, vegetable fats, and breakfast cereals) exhibit better cognitive function than subjects who have a lower intake of healthy diets [29].

It is expected that Asian countries will go through a rapid rise in their ageing populations compared to developed countries [30, 31]. Nevertheless, there is little information available about the risk factors and the incidence rates of MCI. In fact, some local studies showed that risk factors for MCI may be different among older multi-ethnic Asian adults in Malaysia. Differences may also be caused by geographical background, age, study methodology and the definition of decline in cognitive function [32]. Predictors of MCI among older men in Malaysia were married status, lack of exercise, overweight and hypercholesterolemia [33]. Another cross-sectional study indicated that higher fasting blood sugar, hyperlipidaemia, disability, lower education level, lack of regular involvement in technical-based activities and limited use of modern technologies were risk factors for MCI. The same study found that lower intake of fruits and fresh fruit juices and a failure to engage in calorie restriction were among the risk factors of MCI among older adults in Malaysia [34]. This information is vital for developing public health strategies that will promote healthy longevity. Thus, the aim of this study was to obtain an estimate of the incidence rate of MCI and to determine possible MCI predictors among older multi-ethnic Asian adults in Malaysia during a 1½-year follow-up.

## Methods

### Study design and participants

**Fig. 1:**
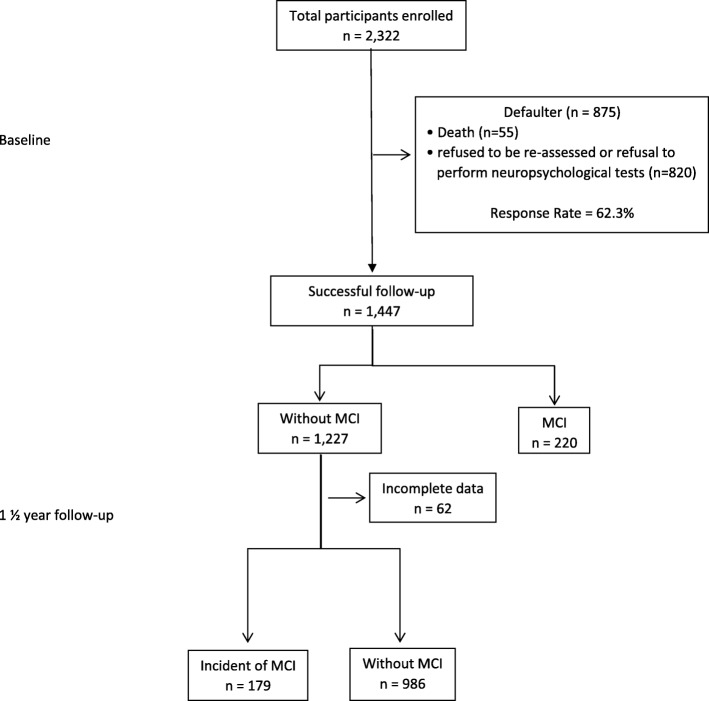
Illustration of the number of participants from baseline to the 1½- year follow-up for MCI incidence

As reported in an earlier report, 2322 older adults consisting of 1208 women and 1114 men were enrolled via a multistage random sampling process. These participants came from four states that represent the southern, northern, central, and eastern regions of Malaysia and that have the highest population of older adults [35]. Inclusion criteria were individuals aged 60 years and above with no known dementia or any other psychiatric problems, no severe vision or auditory-related difficulties and minimal functional limitations (not wheel-chair bound or bed-ridden). Participants were excluded from the study if they had documented evidence of psychiatric disorders. Those participants with the Malay-Mini Mental State Examination (M-MMSE) score of 14 and below were excluded because this indicated moderately severe or severe cognitive impairment. In this study, we selected participants who did not have MCI during baseline. The incidence of MCI referred to MCI development during the 1½-year follow-up, which included having no dementia. Out of the total population, 1447 participants had a successful follow-up after 1½ years (response rate 62.3%). After the 1½-year follow-up, 55 participants had died, and 820 participants refused to be reassessed or refused to undergo neuropsychological testing. Of the participants who went through the follow-up, the analysis included 1227 participants (no MCI at baseline) (Fig. 1). This study was approved by the Research Ethical Committee of the University Kebangsaan Malaysia (LRGS TUA-NN-060-2013). Furthermore, written information was provided, and written consent was obtained from all participants.

### Data collection

Participants underwent interviews by trained enumerators who utilised a structured questionnaire at their respective community centres. The questionnaire consisted of 8 items: socio-demography, neuropsychological and psychosocial functions, lifestyle, dietary intake, anthropometry, blood pressure, physical fitness and functional status. Measurement of all the outcomes was performed at baseline and during the 1½-year (18 months) follow-up period. The factors evaluated were lifestyle (nutrition, smoking, social, physical and mental activities, and physical performance), cognitive functions (assessed with cognitive batteries that include M-MMSE, Digit Span, Montreal Cognitive Assessment (MoCA), Rey Auditory Verbal Learning Test (RAVLT), Digit Symbol, Visual Reproduction Test (VR-I and VR II)), and medical conditions. Because these factors could be dependent on an individual’s cognitive status, participants were stratified at baseline based on the MCI status.

### Incidence of MCI

The incidence of MCI referred to MCI development during the 1½-year follow-up of the participants who did not exhibit MCI at the baseline.

### i) MCI

Participants were classified as having MCI if they satisfied the criteria set by Petersen [36] and Lee et al. [33], which included no evidence of dementia, objective memory impairment (at least 1.5 SD below the mean for either RAVLT or digit span), subjective memory complaint by caregivers or participants, no limitations experienced in basic activities of daily living (ADL), preserved global function evidenced by M-MMSE score of ≥19, and no or extremely minimal difficulties in instrumental activities of daily living evidenced by a score of ≤1.5 SD from the mean norm.

### Possible predictors of MCI

As previously stated, a few variables were studied [34, 35]. Possible predictors of MCI (Table 1) were explained in previously published literature [36].

**Table 1:** Tool used to assess the possible predictors of MCI

Tool	Parameters
Socio-demography	Gender, age, ethnicity, education level, marital status, living arrangement and medical history.
Nutritional status and clinical profile	Weight, height, arm span, mid-upper arm circumference (MUAC), waist circumference, hip circumference, calf circumference, fat mass, muscle mass and blood pressure.
Cognitive function test	M-MMSE, MoCA, Digit Span Test, Digit Symbol Test, RAVLT, VR-I and VR II.
Lifestyle	Lifestyle activities (Victoria Longitudinal Study-Activities Lifestyle Questionnaire) and smoking status.
Physical performance test	Grip Strength Test, Chair Stand Test, Chair Sit-and-Reach Test, Back Scratch Test, Timed Up and-Go Test (TUG).
Dietary intake	Diet history questionnaire (DHQ).
Depression	Geriatric depression scale (GDS-15).

### Statistical analyses

The calculation of the cumulative incidence of MCI was performed by dividing the number of new MCI cases during the follow-up period by the number of at-risk participants in the population at the start of the study. The calculation of the incidence rates of MCI was performed by dividing the number of new MCI cases by the total person-time that was detected between the two evaluations. The number of at-risk participants and total person-time referred to participants with no MCI specifically at baseline (e.g., no MCI, no cognitive impairment and no dementia). The characteristics of the participants were presented using descriptive statistics and were based on the MCI status at 18 months. Those studies have a population with mean μ and standard deviation σ and take sufficiently large random samples from the population with replacement; then, the distribution of the sample means will be approximately normally distributed based on the central limit theorem [37]. Descriptive statistics were used in the analysis of demography, nutritional status, health conditions, neurocognitive, lifestyle and dietary intake assessments. The characteristics of the participants were presented using an independent t-test for numerical data and a χ2-test for categorical data. Quantitative data were expressed as the means ± S.D. According to various categorical variables, the Pearson χ2 test was used to compare differences based on the MCI status at 18 months. Dietary intake assessment for men and women was analysed using an independent t-test, which was carried out to compare mean differences in dietary intake based on the MCI status at 18 months. Identification of the variables that had significant relationships with the incidence of MCI was performed using logistic regression analysis. This process begins with univariate analyses, where the effect of each variable is separately reviewed. It is then followed by binary logistic analyses. The dependent variable used was the MCI status group at 18 months, with no MCI (0 – no MCI, 1 – MCI) as the reference variable. For each model, all the covariates (sex, age, and total educational years) were taken as control variables. Significant values were specified at *p* < 0.05. Other variables which were not significant were also considered as independent variables in the binary logistic analysis, if they were reported as predictors of MCI based on previous literatures.

Binary logistic analyses were performed in two stages. For the first stage, all the significant variables in the univariate analysis were classified into five different groups as follows: socio-demography and nutritional status, fitness, dietary intake, quality of life, and lifestyle. Next, separate binary logistic analyses were conducted for the five models. Variables that appeared significant (*p* < 0.05) in each binary logistic model were selected to be entered in the final binary logistic model. It should be useful to implement the analysis with a bivariate model among significant variables. However in certain case, few variables which found to be non significant such as “attending or organizing parties” [38] and “gardening or rearing animals” [39] has been included in the analysis because they have been reported as protective factor against MCI based on previous literature. Only the final model was shown in this article (Table 5). SPSS version 23.0 (Licensed materials – Property of SPSS, Incorporation, an IBM Company Copyright 1989 and 2010 SPSS) was used to perform these analyses. Additional file 1: Table S1a to S1e show the five models of the binary logistic analysis.

## Results

In this study, 1227 (84.8%) out of the 1447 participants were living on their own and did not have MCI at baseline (Table 2). After conducting the 1½-year follow-up for those who did not have MCI at baseline, 986 (80.4%) remained without MCI, 179 (14.6%) developed MCI, and 62 (5.1%) had incomplete data. In this group, the 1½-year cumulative incidence of MCI was 14.6%. The observed incidence rate was 10.5 per 100 person-years.

**Table 2:** Baseline attributes of the participants according to MCI status at 18 months [presented as the mean (sd) or *n* (%)]

Parameters	*N* = 1227		*p* Value
	MCI (*n* = 179)	Without MCI (*n* = 986)	
Age, mean (sd):	69.2 (5.9)	68.1 (5.8)	0.026*
Sex			
Men	101 (17.6)	473 (82.4)	0.042*
Women	78 (13.2)	513 (86.8)	
Ethnic			
Malay	111 (14.5)	655 (85.5)	0.266
Non-Malay	68 (17.0)	331 (83.0)	
Education (years)			
≤ 6 years	146 (16.9)	716 (83.1)	0.012*
> 6 years	33 (10.9)	270 (89.1)	
Smoking			
Smoker	58 (16.2)	299 (83.8)	0.597
Non-smoker	121 (15.0)	687 (85.0)	
Living alone	22 (18.0)	100 (82.0)	0.425
Body Mass Index			
< 22 kg/m^2^	35 (14.1)	214 (85.9)	0.817
22–27 kg/m^2^	96 (15.8)	513 (84.2)	
> 27 kg/m^2^	47 (15.5)	257 (84.5)	
% Body Fat, mean (sd)	39.6 (9.8)	39.1 (10.3)	0.580
Blood Pressure			
Diastolic (mmHg), mean (sd)	77.2 (13.0)	77.5 (13.5)	0.769
Systolic (mmHg), mean (sd)	142.3 (24.2)	140.1 (22.3)	0.246
Calf Circumference (cm), mean (sd)	33.3 (3.5)	33.5 (3.9)	0.559
Waist Circumference (cm), mean (sd)	88.5 (10.8)	88.0 (11.5)	0.550
MUAC (cm), mean (sd)	28.6 (3.0)	28.6 (3.6)	0.881
TUG (seconds), mean (sd)	11.3 (2.7)	11.3 (3.7)	0.953
Grip Strength (kg), mean (sd)	22.4 (7.7)	22.5 (7.4)	0.880
GDS, mean (sd)	2.5 (2.3)	2.3 (2.0)	0.256
Health Conditions			
Diabetes	45 (15.5)	246 (84.5)	1.000
Hypertension	91 (16.1)	473 (83.9)	0.516
Heart Disease	22 (18.6)	96 (81.4)	0.284
Lung Disease	2 (22.2)	7 (77.8)	0.635
Stroke	3 (14.3)	18 (85.7)	1.000
Neurocognitive			
M-MMSE, mean (sd)	23.0 (4.2)	23.5 (4.7)	0.173
Span Digit, mean (sd)	7.5 (2.4)	7.7 (2.4)	0.326
RAVLT BL, mean (sd)	36.1 (8.4)	40.2 (10.3)	< 0.001*
RAVLT DR, mean (sd)	37.3 (8.3)	41.1 (9.8)	< 0.001*
Lifestyle			
Physical activity, mean (sd)	9.4 (4.1)	10.0 (4.3)	0.068
Social activity, mean (sd)	15.0 (5.1)	15.7 (5.6)	0.143
Mental activity, mean (sd)	12.4 (4.2)	13.6 (4.7)	0.002*

Using independent t-test and χ2-test

*p < 0.05 (significant)

As stated in Table 2, those in the MCI group were significantly (*p* < 0.05) older (69.2 ± 5.9 years old) than participants who did not have MCI (68.1 ± 5.8 years old). Generally, men had a significantly higher risk (*p* < 0.05) of developing MCI (17.6%) than women (13.2%). The MCI group had significantly (p < 0.05) lower education attainment (16.9%) than the non-MCI group (10.9%). The non-MCI group had higher scores in the Rey Auditory Verbal Learning Test Delayed Recall (RAVLT DR), (40.2 ± 10.3 vs 36.1 ± 8.4) and Rey Auditory Verbal Learning Test Total Learning (RAVLT BL), (41.1 ± 9.8 vs 37.3 ± 8.3) (p < 0.05 for both parameters) than the MCI group, respectively. In addition, the non-MCI group reported significantly higher mental activity scores (13.6 ± 4.7) than the MCI group (12.4 ± 4.2) (p < 0.05).

These analyses were focused on determining participants’ nutrient intake per day and fulfilling the percentage of Recommended Nutrient Intakes (RNIs) for Malaysia according to sex. Analysis of nutrient intake was conducted separately for men and women participants because their energy and nutrient requirements differ. Tables 3 and 4 show the nutrient intake of men and women, respectively, as compared to the RNIs for Malaysia. Table 3 shows that the nutrient intake at baseline did not differ significantly between men with and those without MCI at 18 months. However, thiamine intake was significantly higher in women who did not have MCI (1.2 ± 2.5 mg/day) compared to women who developed MCI at 18 months (0.8 ± 0.9 mg/day) (*p* < 0.05) (Table 4).

**Table 3:** Dietary intake among men with MCI and without MCI at 18 months (presented as mean (sd))

Nutrient	*N* = 1227				*p* value
	MCI (*n* = 95)		Without MCI (*n* = 449)		
	Intakes	% of intake from RNIs^a^	Intakes	% of intake from RNIs	
Energy (kcal)	1817 ± 539.2	90.4 ± 26.8	1779 ± 487.0	88.5 ± 24.2	0.528
Protein (g/day)	75.9 ± 23.8	128.6 ± 40.4	74.4 ± 22.4	126.0 ± 37.9	0.553
Protein (% of energy)	16.8 ± 3.0	–	16.9 ± 3.4	–	0.833
Carbohydrate (g/day)	255.3 ± 88.7	–	248.9 ± 79.9	–	0.489
Carbohydrate (% of energy)	55.9 ± 7.4	–	55.7 ± 7.5	–	0.839
Fat (g/day)	54.6 ± 20.2	–	53.7 ± 19.2	–	0.702
Fat (% of energy)	27.2 ± 6.1	–	27.2 ± 6.6	–	0.902
Saturated Fat (g/day)	9.0 ± 5.6	–	9.7 ± 6.5	–	0.330
Total Fibre (g/day)	3.7 ± 2.3	–	3.9 ± 2.5	–	0.397
Vitamin C (mg/day)	120.3 ± 93	171.9 ± 132.8	117.0 ± 90	167.1 ± 129.2	0.745
Vitamin E (mg/day)	23.6 ± 110	235.7 ± 1095	6.1 ± 21.0	60.5 ± 210.3	0.124
Thiamine (mg/day)	1.4 ± 3.2	120.0 ± 266	1.4 ± 3.4	118.0 ± 287	0.949
Riboflavin (mg/day)	1.3 ± 0.5	97.5 ± 41.4	1.2 ± 0.5	95.0 ± 37.0	0.556
Niacin (mg/day)	10.6 ± 3.9	66.0 ± 24.5	10.9 ± 4.1	68.1 ± 25.8	0.459
Pyridoxine (mg/day)	0.7 ± 0.3	–	0.8 ± 0.4	–	0.629
Folate (μg /day)	110.1 ± 74	27.5 ± 18.6	104.1 ± 70	26.0 ± 17.5	0.452
Calcium (mg/day)	548.9 ± 241	68.6 ± 30.1	524.3 ± 239	65.5 ± 29.9	0.365
Sodium (mg/day)	1459 ± 819	–	1575 ± 1097	–	0.330
Iron (mg/day)	14.2 ± 5.9	101.7 ± 42.1	13.9 ± 5.1	99.4 ± 36.4	0.580
Zinc (mg/day)	3.9 ± 2.0	58.6 ± 30.1	3.8 ± 2.1	56.6 ± 30.8	0.554
Phosphorus (mg/day)	1193 ± 415	–	1183 ± 429	–	0.832
Selenium (μg/day)	23.0 ± 19.9	69.6 ± 60.2	22.8 ± 17.9	69.0 ± 54.2	0.923

^a^RNIs: Recommended Nutrient Intakes for Malaysia [40]

**Table 4:** Dietary intake among women with MCI and without MCI at 18 months (presented as mean (sd))

Nutrient	*N* = 1227				*p* value
	MCI (*n* = 69)		Without MCI (*n* = 494)		
	Intakes	% of intake from RNIs^a^	Intakes	% of intake from RNIs	
Energy (kcal)	1563 ± 465.6	87.9 ± 26.2	1506 ± 430.3	84.6 ± 24.2	0.299
Protein (g/day)	68.9 ± 25.8	135.0 ± 50.5	66.5 ± 22.1	130.3 ± 43.3	0.412
Protein (% of energy)	17.7 ± 3.6	–	17.8 ± 3.8	–	0.848
Carbohydrate (g/day)	206.7 ± 66.6	–	196.9 ± 64.1	–	0.239
Carbohydrate (% of energy)	53.1 ± 8.1	–	52.4 ± 8.4	–	0.531
Fat (g/day)	51.3 ± 21.4	–	50.2 ± 19.8	–	0.679
Fat (% of energy)	29.2 ± 6.8	–	29.8 ± 7.0	–	0.488
Saturated Fat (g/day)	7.4 ± 5.0	–	7.6 ± 5.4	–	0.769
Total Fibre (g/day)	3.6 ± 2.0	–	4.1 ± 2.4	–	0.084
Vitamin C (mg/day)	112.3 ± 84.0	160.4 ± 120.0	118.0 ± 83.5	168.6 ± 119.3	0.594
Vitamin E (mg/day)	14.3 ± 60.0	190.4 ± 800	18.4 ± 88.9	244.8 ± 1185	0.712
Thiamine (mg/day)	0.8 ± 0.9	76.3 ± 85	1.2 ± 2.5	113.5 ± 225	0.011*
Riboflavin (mg/day)	1.1 ± 0.5	103.6 ± 48.3	1.2 ± 0.5	109.8 ± 46.6	0.299
Niacin (mg/day)	9.9 ± 4.5	70.5 ± 32.2	9.9 ± 3.7	70.6 ± 26.7	0.989
Pyridoxine (mg/day)	0.6 ± 0.3	–	0.7 ± 0.3	–	0.174
Folate (μg /day)	92.8 ± 56.7	23.2 ± 14.2	107.1 ± 78	26.8 ± 19.4	0.139
Calcium (mg/day)	458.6 ± 199	45.9 ± 19.9	505.2 ± 250	50.5 ± 25.0	0.139
Sodium (mg/day)	1437 ± 823	–	1311 ± 956	–	0.295
Iron (mg/day)	12.8 ± 5.5	116.3 ± 50.2	12.9 ± 5.2	117.6 ± 47.6	0.831
Zinc (mg/day)	3.0 ± 1.6	61.5 ± 32.1	3.4 ± 1.8	69.0 ± 36.7	0.107
Phosphorus (mg/day)	1028 ± 369	–	1010 ± 402	–	0.714
Selenium (μg/day)	26.3 ± 17.1	105.0 ± 68.4	23.9 ± 17.8	95.4 ± 71.3	0.292

^a^RNIs: Recommended Nutrient Intakes for Malaysia [40]

The findings revealed that among men with and without MCI at 18 months, the mean energy and nutrient intakes were below the RNIs for Malaysia, except for vitamin C, protein, and thiamine (Table 3). Furthermore, among men with MCI at 18 months, the mean intake of iron and vitamin E met the RNIs, while iron and vitamin E intake among those without MCI at 18 months was below the RNIs. On the other hand, among women with and without MCI at 18 months, the mean energy and nutrient intake were below the RNIs, except for vitamin C, protein, riboflavin, vitamin E, and iron (Table 4).

The binary logistic regression models were stratified for non-MCI at baseline, with potential confounders and predictors defined as independent variables (Table 5). Based on the binary logistic regression analysis that was conducted for non-MCI subjects during baseline, male sex (adjusted OR = 1.854; CI = 1.287–2.672; *p* = 0.001) had a significant relationship to MCI during the follow-up. Furthermore, a greater number of mental activities (adjusted OR = 0.936; CI = 0.897–0.977; *p* = 0.002) reduced the risk of MCI among persons who did not have MCI at baseline. Other variables did not exhibit any significant relationship with MCI in this study.

**Table 5:** Potential predictors for MCI at 18 months

Predictor of interest	*N* = 1227		*p* value
	B	OR (95% CI)	
Age (years, continuous)	0.019	1.019 (0.987–1.052)	0.245
Sex			
Male vs Female	0.618	1.854 (1.287–2.672)	0.001*
Education (years):			
0–6 vs 7 and above	0.415	1.515 (0.952–2.409)	0.079
Lifestyle			
Mental activity	– 0.066	0.936 (0.897–0.977)	0.002*
Attend or organise parties	0.389	1.476 (0.860–2.534)	0.158
Gardening or rearing animals	0.184	1.201 (0.850–1.699)	0.299
Use modern gadgets	0.532	1.702 (0.619–4.681)	0.303
Physical Performance			
TUG ((seconds)	– 0.030	0.971 (0.919–1.026)	0.294
Dietary Intake:			
Pyridoxine (mg/day)	– 0.249	0.779 (0.470–1.292)	0.333

*p < 0.05 significant using binary logistic regression

## Discussion

In the Asian region, the prevalence of MCI in older adults has mostly been described in cross-sectional studies. However, there is very limited available information on the incidence of MCI in prospective studies [6]. Based on the results of this study, the incidence rate of MCI was 10.5 per 100 person-years for older adults who did not exhibit MCI at baseline. This rate was higher compared to previous reports in China (i.e., 2.3% per year) [41], Italy (i.e., 2.15 per 100 person-years) [42] and Hong Kong (i.e., 6.37 per 100 {women}) [6]. Nevertheless, one should note that the incidence rates obtained in the studies conducted in Hong Kong, Italy, and China were measured at 3, 3.5 and 5 years of follow-up, respectively, while the present study reported results from a 1½-year follow-up period. A longer time frame for the follow-up needs to be established so that the trend for the incidence rate of MCI can be better determined. Discrepancies in the cognitive incidence rate could be attributed to several variations, including follow-up time, geographic settings, age of participants, data collection, method and sample size, operational definition of cognitive decline, and the cognitive batteries utilised [4].

In general, the results of this study suggest that lifestyle (mental activities) and sex were vital MCI predictors among multi-ethnic Asian populations. Among older Malaysian adults, mental activities were found to be predictors of MCI, an observation that was also made in an earlier study [34]. Compared to women, the present study observed that men had twice the risk of MCI. This result coincides with the results from the Sydney Memory and Ageing Study [4], which reported that men had higher MCI incidence rates, as observed in 889 community-dwelling individuals aged 70–90 years after a 2-year follow-up period. According to another longitudinal study that was conducted among older Italian adults, the global incidence rates of MCI among men were not shown to be lower than those in women [42].

Among men, the risk of cognitive impairment was increased with poor social support and divorce status [6]. Another study [43] reported that sex-related differences in exposure of social activities may influence in mental status [44]. Consequently, a study conducted among older Chinese adults in Hong Kong revealed that being female was an independent factor related to cognitive impairment [6]. It has also been reported that loss of work-related roles (due to retirement or job loss) threatens the mental health of men more than the mental health of women [45]. Furthermore, men are likely to experience more distress than women after losing a spouse, mainly due to their lack of social networks and support and the burden of having to perform domestic tasks [46]. Living alone is also especially damaging to men [47]. These are possible explanations for the findings of our study.

In this study, the specific domain of the lifestyle related to mental activity had an inverse relationship with MCI. For participants who did not have MCI at baseline, increasing mental activity by one unit lowered the risk of cognitive decline by 10% during the 1½-year follow-up. These results coincide with previous studies [48–50], showing that a boost in mental activities such as doing jigsaw puzzles, playing chess, reading magazines, newspapers, and books, watching television, and playing cards were associated with delays in the development of MCI and decline in cognitive function. Similarly, according to a longitudinal study conducted in Switzerland, increased involvement in mental activities (listening to the radio, watching television, reading books, newspapers, or magazines) was observed to slow the decline in cognition [51]. According to another study [49], mental activity is related to enhanced memory, language, executive function, and cognitive skills and reduced perception of speed reduction [51]. Thus, any mental activity that includes the process of thinking and attention control could maintain or even increase the brain reserve even in advanced age [52, 53]

Furthermore, reports from systematic reviews that encompass both intervention and observational studies revealed that increased mental activity protects cognitive function [18]. Frequently engaging in mental activity may contribute to functional and structural neural reorganisation, which lowers the vulnerability of neurons to destruction from Alzheimer’s disease-related pathology [49]. It has also been stated that constant engagement in mentally stimulating activities promotes stability or improves cognitive performance [52]. Furthermore, it has been observed that mental activities increase cellular activity and cerebral blood flow in the brain and also increases the metabolic rate of two vital brain networks that regulate the connectivity of higher order cognitive control processes [54].

In the current study, it was determined that being involved in mental activities (e.g., utilising modern technology) protects against the incidence of MCI among the 1227 older adults who did not have MCI at baseline. This result agrees with the study performed among 2611 individuals in the Chinese community in Singapore, which found that increasing mental activity by activities such as listening to music and reading is associated with decreased risks of cognitive decline [55]. Moreover, an RCT observed that cognitive functions of older adults who attended computer classes were positively affected [56]. Improved cognition has also been associated with mental activity engagement among older males in China [39]. In a similar manner, a prospective study conducted with 469 older adults with no dementia found that being involved in cognitive stimulation activities (e.g., playing board games) served as protection against the onset of dementia [57]. This mental stimulation process could have a role in maintaining cognition [58].

The current study possesses several strengths. First, this study utilised six domains for the measurement of cognitive function (M-MMSE, Digit Span, MoCA, Digit Symbol, VR-I and VR II, and RAVLT) based on their categorisation of cognitive decline. Second, lifestyle-related factors (smoking, nutrition, physical performance, physical, mental, and social activities) were also assessed for their role as predictors of MCI. Third, this present study is classified as a longitudinal study that has a large sample size. However, there are some limitations that can affect studies that evaluate the incidence and predictors of MCI. First, there is the short follow-up period in our longitudinal study. Future studies will require a longer follow-up period to better assess the incidence of MCI. Second, there is a lack of a specific tool for MCI diagnosis, especially in subjects who refuse to answer survey questions. The other limitation is that the heterogeneity of samples also affects our longitudinal study due to differences in origins, locations and cultures.

## Conclusion

To conclude, among the older adults in Malaysia, the incidence rate of MCI was 10.5 per 100 person-years for individuals who did not have MCI at baseline. Being male and having less engagement in mental activities were determined to be MCI predictors among participants who did not have MCI at baseline. After a short follow-up period, the high incidence rate observed for MCI is indicative of the need to implement a holistic approach. However, these findings offer preliminary support for intervention studies in the future that will work towards the optimisation of the cognitive health of older adults. Furthermore, effective preventive management strategies need to be formulated to slow the rate of cognitive decline leading to the occurrence of MCI among older adults.

## Additional file

Additional file 1: **Table S1a to S1e.** Five models of the binary logistic analysis. (DOCX 21 kb)

## Data Availability

Not applicable.

## Abbreviations

ADL: Activities of Daily Living
DHQ: Diet History Questionnaire
GDS: Geriatric Depression Scale
MCI: Mild Cognitive Impairment
M-MMSE: Malay-Mini Mental State Examination
MoCA: Montreal Cognitive Assessment
MUAC: Mid-Upper Arm Circumference
RAVLT BL: Rey Auditory Verbal Learning Test Total Learning
RAVLT DR: Rey Auditory Verbal Learning Test Delayed Recall
RAVLT: Rey Auditory Verbal Learning Test
RNIs: Recommended Nutrient Intakes
TUA: Towards Useful Aging
TUG: Timed Up and-Go Test
VR-I and VR II: Visual Reproduction Test

## References

[1] Gauthier S, Reisberg B, Zaudig M, Petersen RC, Ritchie K, Broich K, Belleville S, Brodaty H, Bennett D, Chertkow H, Cummings JL, Feldman H, Ganguli M, Hampel H, Scheltens P, Tierney MC, Whitehouse P, Winblad B (2006). Mild cognitive impairment. Lance.

[2] Daviglus ML, Bell CC, Berrettini W, Bowen PE, Connolly ES, Jr Cox NJ, Dunbar-Jacob JM, Granieri EC, Hunt G, McGarry K, Patel D, Potosky AL, Sanders-Bush E, Silberberg.D, Trevisan M: NIH State-Of-The-Science Conference Statement: Preventing Alzheimer's Diseases and Cognitive Decline. NIH Consens State Sci Statements 2010, 28, 27(4): 1–30. 20445638

[3] Zuria Idura AM, Noorlaili MT, Rosdinom R, Azlin B, Iryani T (2018). Caring for moderate to severe dementia patients- Malaysian family caregivers experience. International Medical Journal Malaysia.

[4] Lipnicki DM, Sachdev PS, Crawford J, Reppermund S, Kochan NA, Trollor JN, Draper B, Slavin MJ, Kang K, Lux O, Mather KA, Brodaty H (2013). Risk factors for late-life cognitive decline and variation with age and sex in the Sydney memory and ageing study. PLoS One.

[5] Barnes DE, Yaffe K, Satariano WA, Tager IB (2003). A longitudinal study of cardiorespiratory fitness and cognitive function in healthy older adults. J Am Geriatr Soc.

[6] Ho SC, Woo J, Sham A, Chan G, Lm A (2001). A 3-year follow-up study of social, lifestyle and health predictors of cognitive impairment in a Chinese older cohort. Int J Epidemiol.

[7] Zhang M, Katzman R, Salmon D (1990). The prevalence of dementia and Alzheimer’s disease in Shanghai, China: impact age, gender and education. Ann Neurol.

[8] Gammazza AM, Restivo V, Baschi R, Bavisotto CC. Cefal` AB, Accardi G, de Macario EC, Macario AJL, Cappello F, Monastero R: circulating molecular chaperones in subjects with amnestic mild cognitive impairment and Alzheimer’s disease: data from the Zabùt aging project. J Alzheimers Dis. 2018:1–12. 10.3233/JAD-180825 PMC9277667 30584145

[9] Reinprecht F, Elmstahl S, Janzon L, Andre-Petersson L (2003). Hypertension and changes of cognitive function in 81-year-old men: a 13-year follow-up of the population study “men born in 1914”, Sweden. J Hypertens.

[10] Reitz C, Tang MX, Manly J, Mayeux R, Luchsinger JA (2007). Hypertension and the risk of mild cognitive impairment. Arch Neurol.

[11] Cherbuin N, Reglade-Meslin C, Kumar R (2009). Risk factors of transition from normal cognition to mild cognitive disorder: the PATH through life study. Dement Geriatr Cogn Disord.

[12] Luchsinger JA, Reitz C, Patel B, Tang MX, Manly JJ, Mayeux R (2007). Relation of diabetes to mild cognitive impairment. Arch Neurol.

[13] Ott A, Andersen K, Dewey ME, Letenneur L, Brayne C, Copeland JRM, Dartigues JF (2004). Kragh–Sorensen P, lobo a, Martinez–Lage JM, Stijnen T, Hofman TA, Launer LJ: effect of smoking on global cognitive function in nondemented elderly. Neurology.

[14] Barnes DE, Alexopoulos GS, Lopez OL, Williamson JD, Yaffe K (2006). Depressive symptoms, vascular disease mild cognitive impairment: findings from the cardiovascular health study. Arch Gen Psychiatry.

[15] Paterniti Sabrina, Verdier-Taillefer Marie-Hélène, Dufouil Carole, Alpérovitch Annick (2002). Depressive symptoms and cognitive decline in elderly people. British Journal of Psychiatry.

[16] Wilson R (2004). Mendes d, Bennett D, Bienias J, Evans D: depressive symptoms and cognitive decline in a community population of older persons. J Neurol Neurosurg Psychiatry.

[17] Abdul Manaf MR, Mustafa M, Abdul Rahman MR, Yusof KH, Abd Aziz NA (2016). Factors influencing the prevalence of mental health problems among Malay elderly residing in a rural community: a cross-sectional study. PLoS One.

[18] Wang HX, Xu W, Pei JJ (2012). Leisure activities, cognitive function and dementia. Biochim Biophys Acta.

[19] Saczynski JS, Jonsdottir MKM, Sigurdsson S (2008). White matter lesions and cognitive performance: the role of cognitively complex leisure activity. J Geront A Biol Sci Med Sci.

[20] Dodge H. H., Kita Y., Takechi H., Hayakawa T., Ganguli M., Ueshima H. (2008). Healthy Cognitive Aging and Leisure Activities Among the Oldest Old in Japan: Takashima Study. The Journals of Gerontology Series A: Biological Sciences and Medical Sciences.

[21] Tseng CN, Gau BS, Lou MF (2011). The effectiveness of exercise on improving cognitive function in older people: a systematic review. J Nurs Res.

[22] Carvalho A, Rea IM, Parimon T, Cusack BJ. Physical activity and cognitive function in individuals over 60 years of age: a systematic review. Clin Interv Aging. 2014:661–81. 10.2147/CIA.S55520 PMC3990369 24748784

[23] Weuve J, Kang JH, Manson JE, Breteler MMB, Ware JH, Grodstein F (2004). Physical activity, including walking, and cognitive function in older women. JAMA.

[24] Poh BK, Safiah MY, Tahir A, Siti Haslinda MD, Siti Norazlin N, Farina Z, Mohd Hasyami S: Physical activity of adults aged 18 to 59 years, *Malaysian Adult Nutrition Survey 2003*. Volume 6. Putrajaya: Putrajaya; 2008.

[25] Poh BK, Safiah MY, Tahir A, Siti Haslinda MD, Siti Norazlin N, Norimah AK, Wan Manan WM, Mirnalini K, Zalilah MS, Azmi MY, Fatimah S (2010). Physical activity pattern and energy expenditure of Malaysian adults: findings from the Malaysian adult nutrition survey (MANS). Mal J Nutr.

[26] Ibrahim S, Karim AN, Onn NL, Wan Ngah WZ (2013). Perceived physical activity barriers related to body weight status and sociodemographic factors among Malaysian men in Klang Valley. BMC Public Health.

[27] Qiu C, Kivipelto M, Fratiglioni L (2011). Preventing Alzheimer disease and cognitive decline. Ann Intern Med.

[28] Tangney CC, Kwasny MJ, Li H, Wilson RS, Evans DA, Morris MC (2011). Adherence to a Mediterranean-type dietary pattern and cognitive decline in a community population. Am J Clin Nutr.

[29] Kesse-Guyot E, Andreeva VA, Jeandel C, Ferry M (2012). Hercberg S & Galan P: “a healthy dietary pattern at midlife is associated with subsequent cognitive performance”. J Nutr.

[30] Cooper C, Campion G, Melton LJ (1992). Hip fractures in the elderly: a world-wide projection. Osteoporosis Int.

[31] United Nations: The Aging of Asian Population Department for Economic and Social Info and Policy Analysis 2013. New York, USA.

[32] Bischkopf J, Busse A, Angermeyer MC (2002). Mild cognitive impairment – a review of prevalence, incidence and outcome according to current approaches. Acta Psychiatr Scand.

[33] Lee LK, Shahar S, Chin AV, Mohd Yusoff NA, Rajab NF, Abdul Aziz S (2012). Prevalence of gender disparities and predictors affecting the occurrence of mild cognitive impairment (MCI). Arch Gerontol Geriatr.

[34] Divya V, Suzana S, Normah CD, Azahadi O, Chin AV, Rosdinom R (2017). Predictors of poor cognitive status among older Malaysian adults: baseline findings from the LRGS TUA cohort study. Aging Clin Exp Res.

[35] Shahar S, Omar A, Vanoh D, Hamid TA, Mukari SZM, Che Din N (2015). Approaches in methodology for population-based longitudinal study on neuroprotective model for healthy longevity (TUA) among Malaysian older adults. Aging Clin Exp Res.

[36] Hussin NM, Shahar S, Din NC, Singh DKA, Chin AV, Razali R, Omar MA (2019). Incidence and predictors of multimorbidity among a multiethnic population in Malaysia: a community-based longitudinal study. Aging Clin Exp Res.

[37] Endgames: Statistical question: Very large sample sizes. BMJ 2009, 338: b737.

[38] Pillemer SC, Holtzer R (2016). The differential relationships of dimensions of perceived social support with cognitive function among older adults. Aging Ment Health.

[39] Wang HX, Jin Y, Hendrie HC, Liang C, Yang L, Cheng Y, Unverzagt FW, Ma F, Hall KS, Murrell JR, Li P, Bian J, Pei JJ, Gao S (2013). Late life leisure activities and risk of cognitive decline. J Gerontol A Biol Sci Med Sci.

[40] National Coordinating Committee on Food and Nutrition: Recommended Nutrient Intakes for Malaysia (RNI). A Report of the Technical Working Group on Nutritional Guidelines 2005. 1st ed. Ministry of Health Malaysia, Putrajaya.

[41] Wang JY, Zhou DH, Li J, Zhang M, Deng J, Tang M, Gao C, Lian Y, Chen M (2006). Leisure activity and risk of cognitive impairment: the Chongqing aging study. Neurology.

[42] Solfrizzi V, Panza F, Colacicco AM (2004). Vascular risk factors, incidence of MCI, and rates of progression to dementia. Neurology.

[43] Kim KW, Park JH, Kim MH, Kim MD, Kim BJ, Kim SK (2011). A nationwide survey on the prevalence of dementia and mild cognitive impairment in South Korea. J Alzheimers.

[44] Jeon GS, Jang SN, Rhee SJ, Kawachi I, Cho SI (2007). Gender differences in correlates of mental health among elderly Koreans. J Gerontol B Psychol Sci Soc Sci.

[45] Moller-Leimkuhler AM (2003). The gender gap in suicide and premature death or: why are men so vulnerable?. Eur Arch Psychiatry Clin Neurosci.

[46] Lee GR, DeMaris A, Bavin S, Sullivan R (2001). Gender differences in the depressive effect of widowhood in later life. Journal of Gerontology: Social Sciences.

[47] Joutsenniemi K, Martelin T, Martikainen P, Pirkola S, Koskinen S (2006). Living arrangements and mental health in Finland. Journal of Epidemiology and Community Health.

[48] Bosma H, Van Boxtel MP, Ponds RW (2002). Engaged lifestyle and cognitive function in middle and old aged, non-demented persons: a reciprocal association?. Z Gerontol Geriatr.

[49] Wilson RS, Bennet DA, Bienias JL (2003). Cognitive activity and cognitive decline in a biracial community population. Neurology.

[50] Verghese J, LeValley A, Derby C (2006). Leisure activities and the risk of amnestic mild cognitive impairment in the elderly. Neurology.

[51] Ghisletta P, Bickel JF, Lovden M (2006). Does activity engagement protect against cognitive decline in old age? Methodological and analytical considerations. J Gerontol B Psychol Sci Soc Sci.

[52] Hultsch DF, Hammer M, Small BJ (1993). Age differences in cognitive performance in later life: relationships to self-reported health and activity life style. J Gerontol.

[53] Schooler C (1984). Psychological effects of complex environments during the life span: a review and theory. Intelligence.

[54] Chapman SB, Aslan S, Spence JS, Hart JJ, Bartz EK, Didehbani N, Keebler MW, Gardner CM, Strain JF, DeFina LF, Lu H (2015). Neural mechanism of brain plasticity with complex cognitive training in healthy seniors. Cereb Cortex.

[55] Niti M, Yap KB, Kua EH (2008). Physical, social and productive leisure activities, cognitive decline and interaction with APOE-epsilon 4 genotype in Chinese older adults. Int Psychogeriatr.

[56] Klusmann V, Evers A, Schwarzer R (2010). Complex mental and physical activity in older women and cognitive performance: a 6-month randomized controlled trial. J Gerontol A Biol Sci Med Sci.

[57] Verghese J, Lipton RB, Katz MJ (2003). Leisure activities and the risk of dementia in the elderly. N Engl J Med.

[58] Wang HX, Karp A, Winblad B, Fratiglioni L (2002). Late-life engagement in social and leisure activities is associated with a decreased risk of dementia: a longitudinal study from the Kungsholmen project. Am J Epidemiol.

