# Supplementary material for: Incidence and predictors of mild cognitive impairment (MCI) within a multi-ethnic Asian populace: a community-based longitudinal study
Source: BMC Public Health. 2019 Aug 22;19:1159. doi: 10.1186/s12889-019-7508-4 (PMC6704715; doi:10.1186/s12889-019-7508-4)
Supplement: Supplementary file 1 — Table S1a to S1e. Five models of the binary logistic analysis. (DOCX 21 kb) [file 12889_2019_7508_MOESM1_ESM.docx]

**Five Models of Binary Logistic**

**Table 1a**  Effect of socio-demography, anthropometry, physical performance and clinical factor

of incidence MCI at 18 months. [Present as OR (95% CI)], [N = 1,227], **(Model 1)**.

| Predictor | B | OR (95% CI) | *p* Value |
| --- | --- | --- | --- |
| Age (years) | 0.003 | 1.003 (0.964 – 1.044) | 0.870 |
| Sex: |  |  |  |
| Men vs Women | 0.638 | 1.893 (1.277 – 2.808) | 0.001* |
| Education (years): |  |  |  |
| 0 – 6 vs 7 years and above | 0.891 | 2.438 (1.473 – 4.036) | 0.001* |
| Anthropometry: |  |  |  |
| Body Mass Index: |  |  |  |
| < 22 kg/m^2^ | – | – | – |
| 22 – 27 kg/m^2^ | 0.143 | 1.154 (0.405 – 3.289) | 0.789 |
| > 27 kg/m^2^ | 0.242 | 1.273 (0.647 – 2.506) | 0.485 |
| % Body Fat | 0.017 | 1.017 (0.996 – 1.038) | 0.109 |
| Calf Circumference (cm) | – 0.040 | 0.961 (0.907 – 1.018) | 0.179 |
| Waist Circumference (cm) | – 0.008 | 0.992 (0.965 – 1.020) | 0.575 |
| MUAC (cm) | 0.040 | 1.041 (0.950 – 1.140) | 0.388 |
| Physical Performance: |  |  |  |
| TUG (seconds) | – 0.073 | 0.930 (0.865 – 0.999) | 0.046* |
| Hand Grip (kg) | – 0.008 | 0.992 (0.956 – 1.028) | 0.654 |
| Chair Stand Test | – 0.029 | 0.972 (0.895 – 1.055) | 0.494 |
| Chair Sit and Reach Test (cm) | – 0.001 | 0.999 (0.981 – 1.017) | 0.894 |
| Back Scratch Test (cm) | 0.009 | 1.009 (0.994 – 1.024) | 0.225 |
| Clinical: |  |  |  |
| Blood Pressure: |  |  |  |
| Diastolic (mmHg) | – 0.008 | 0.992 (0.974 – 1.011) | 0.417 |
| Systolic (mmHg) | 0.002 | 1.002 (0.994 – 1.010) | 0.664 |
| Hemoglobin (g/dL) | 0.030 | 1.030 (0.940 – 1.129) | 0.525 |
| Fasting blood sugar (mmol) | – 0.033 | 0.967 (0.877 – 1.067) | 0.506 |

MUAC, Mid-Upper Arm Circumference

* p < 0.05 significant using binary logistic regression

**Table 1b** Effect of quality of life, lifestyle factor of incidence MCI at 18 months.

[Present as OR (95% CI)], [N = 1,227], **(Model 2)**.

| Predictor | B | OR (95% CI) | *p* Value |
| --- | --- | --- | --- |
| Age (years) | 0.029 | 1.029 (0.999 – 1.060) | 0.057 |
| Sex: |  |  |  |
| Men vs Women | 0.359 | 1.431 (1.021 – 2.006) | 0.037* |
| Education (years): |  |  |  |
| 0 – 6 vs 7 years and above | 0.557 | 1.745 (1.142 – 2.667) | 0.010* |
| Quality of Life: | 0.313 | 1.368 (0.753 – 2.483) | 0.303 |
| Smoking: |  |  |  |
| Smoker vs Non-smoker | – 0.303 | 0.739 (0.484 – 1.127) | 0.160 |
| Living: |  |  |  |
| Alone vs Together | 0.234 | 1.263 (0.750 – 2.128) | 0.380 |
| Depression (GDS) | 0.059 | 1.061 (0.980 – 1.148) | 0.144 |
| Functional Status (IADL) | 0.079 | 1.083 (0.994 – 1.179) | 0.068 |
| Non-Fasting: |  |  |  |
| Non-Fasting vs Fasting | – 0.064 | 0.938 (0.612 – 1.439) | 0.771 |

GDS, Geriatric Depression Scale

IADL, Instrumental Activity Daily Living

* p < 0.05 significant using binary logistic regression

**Table 1c** Effect of lifestyle (activity) factor of incidence MCI at 18 months.

[Present as OR (95% CI)], [N = 1,227], **(Model 3)**.

| Predictor | B | OR (95% CI) | *p* Value |
| --- | --- | --- | --- |
| Age (years) | 0.012 | 1.012 (0.983 – 1.041) | 0.429 |
| Sex: |  |  |  |
| Men vs Women | 0.538 | 1.713 (1.216 – 2.413) | 0.002* |
| Education (years): |  |  |  |
| 0 – 6 vs 7 years and above | 0.332 | 1.394 (0.905 – 2.148) | 0.131 |
| Lifestyle: |  |  |  |
| Physical activity | 0.007 | 1.007(0.966 – 1.050) | 0.730 |
| Social activity | 0.001 | 1.001 (0.968 – 1.035) | 0.948 |
| Mental activity | – 0.074 | 0.928 (0.894 – 0.964) | < 0.001* |

* p < 0.05 significant using binary logistic regression

**Table 1d** Effect of lifestyle (detail activities) factor of incidence MCI at 18 months.

[Present as OR (95% CI)], [N = 1,227], **(Model 4)**.

| Predictor | B | OR (95% CI) | *p* Value |
| --- | --- | --- | --- |
| Age (years) | 0.017 | 1.017 (0.989 – 1.047) | 0.232 |
| Sex: |  |  |  |
| Men vs Women | 0.360 | 1.434 (1.035 – 1.987) | 0.030* |
| Education (years): |  |  |  |
| 0 – 6 vs 7 years and above | 0.240 | 1.271 (0.812 – 1.987) | 0.294 |
| Lifestyle Activities: |  |  |  |
| Driving or riding | 0.267 | 1.306 (0.893 – 1.908) | 0.168 |
| Gardening or rearing animals | 0.281 | 1.325 (0.957 – 1.832) | 0.090 |
| Having meals out | – 0.051 | 0.951 (0.665 – 1.360) | 0.781 |
| Exercising | – 0.062 | 0.940 (0.666 – 1.327) | 0.726 |
| Simple repairing activities | 0.226 | 1.254 (0.505 – 3.116) | 0.626 |
| Visiting friends or relatives | – 0.029 | 0.972 (0.667 – 1.416) | 0.882 |
| Sewing | 0.267 | 1.306 (0.609 – 2.799) | 0.493 |
| Involve in outdoor activities such as fishing, catering, business | – 0.259 | 0.772 (0.431 – 1.384) | 0.385 |
| Helping in household chores | – 0.250 | 0.779 (0.511 – 1.188) | 0.246 |
| Attend or organize parties | 0.479 | 1.615 (0.971 – 2.685) | 0.065 |
| Reading | 0.321 | 1.378 (0.924 – 2.056) | 0.116 |
| Attend workshop, courses or talks | – 0.109 | 0.897 (0.628 – 1.280) | 0.547 |
| Shopping | – 0.035 | 0.966 (0.679 – 1.374) | 0.846 |
| Become member of association | 0.367 | 1.444 (0.848 – 2.460) | 0.176 |
| Watching television | – 0.538 | 0.584 (0.290 – 1.177) | 0.132 |
| Involve in financial activities such as household finance or business | 0.008 | 1.008 (0.699 – 1.454) | 0.965 |
| Deliver talk or involve in teaching activities | 0.382 | 1.465 (0.683 – 3.142) | 0.327 |
| Involve in voluntary activities | – 0.110 | 0.895 (0.420 – 1.907) | 0.775 |
| Use modern gadgets | 1.084 | 2.957 (1.170 – 7.469) | 0.022* |
| Involve in food preparation | 0.067 | 1.069 (0.661 – 1.729) | 0.785 |
| Vacation | – 0.445 | 0.641 (0.250 – 1.643) | 0.355 |
| Child care | – 0.120 | 0.887 (0.610 – 1.288) | 0.528 |
| Rearing pet | 0.017 | 1.018 (0.717 – 1.444) | 0.922 |

* p < 0.05 significant using binary logistic regression

**Table 1e** Effect of dietary intake factor of incidence MCI at 18 months.

[Present as OR (95% CI)], [N = 1,227], **(Model 5)**.

| Predictor | B | OR (95% CI) | *p* Value |
| --- | --- | --- | --- |
| Age (years) | 0.024 | 1.024 (0.995 – 1.054) | 0.102 |
| Sex: |  |  |  |
| Men vs Women | 0.456 | 1.577 (1.117 – 2.229) | 0.010* |
| Education (years): |  |  |  |
| 0 – 6 vs 7 years and above | 0.564 | 1.758 (1.147 – 2.696) | 0.010* |
| Dietary Intake: |  |  |  |
| Energy (kcal) | – 0.001 | 0.999 (0.992 – 1.006) | 0.728 |
| Protein (g/day) | 0.008 | 1.008 (0.999 – 1.017) | 0.070 |
| Fat (g/day) | 0.006 | 1.006 (0.993 – 1.019) | 0.386 |
| Saturated Fat (g/day) | – 0.021 | 0.979 (0.945 – 1.014) | 0.243 |
| Total Fibre (g/day) | – 0.052 | 0.949 (0.875 – 1.029) | 0.207 |
| Sugar (g/day) | 0.007 | 1.007 (0.996 – 1.018) | 0.225 |
| Vitamin E (mg/day) | 0.001 | 1.001 (0.999 – 1.003) | 0.265 |
| Thiamine (mg/day) | – 0.014 | 0.986 (0.923 – 1.054) | 0.686 |
| Riboflavin (mg/day) | – 0.116 | 0.891 (0.544 – 1.459) | 0.646 |
| Niacin (mg/day) | – 0.056 | 0.946 (0.884 – 1.012) | 0.108 |
| Pyridoxine (mg/day) | – 0.608 | 0.544 (0.297 – 0.996) | 0.049* |
| Iron (mg/day) | 0.013 | 1.013 (0.972 – 1.056) | 0.540 |
| Zinc (mg/day) | 0.003 | 1.003 (0.888 – 1.133) | 0.960 |
| Selenium (µg/day) | 0.007 | 1.007 (0.996 – 1.017) | 0.203 |

* p < 0.05 significant using binary logistic regression
